# Myalgic encephalomyelitis/chronic fatigue syndrome (ME/CFS) and fibromyalgia: PR3-versus MPO-ANCA-associated vasculitis, an exploratory cross-sectional study

**DOI:** 10.1016/j.lana.2023.100460

**Published:** 2023-02-27

**Authors:** Charmaine van Eeden, Naima Mohazab, Desiree Redmond, Elaine Yacyshyn, Alison Clifford, Anthony S. Russell, Mohammed S. Osman, Jan Willem Cohen Tervaert

**Affiliations:** a Division of Rheumatology, Department of Medicine, Faculty of Medicine and Dentistry, University of Alberta, Edmonton, Canada; b University of Alberta, Rm5-68, Heritage Medical Research Center, Edmonton, T6G 2S2, Canada; c University of Alberta, 8-130 Clinical Sciences Building, Edmonton, T6G 2B7, Canada

**Keywords:** Fatigue, Myalgic encephalomyelitis/chronic fatigue syndrome, Fibromyalgia, Antineutrophil cytoplasmic antibody (ANCA)-Associated vasculitis, Proteinase 3 (PR3) associated ANCA vasculitis, Myeloperoxidase (MPO) associated vasculitis

## Abstract

**Background:**

Persistent fatigue is a common complaint in ANCA-vasculitis (AAV) patients and has a profound impact on patient's quality of life. The symptoms associated with this fatigue mirror those found in patients with myalgic encephalomyelitis/chronic fatigue syndrome (ME/CFS) and fibromyalgia. Etiologic and pathophysiologic differences exist between PR3- and MPO-ANCA disease, yet differences in their fatigue manifestations have not been well researched. We compared fatigue and its associations in healthy controls, AAV patients and fibromyalgia controls.

**Methods:**

The Canadian consensus criteria were used for ME/CFS diagnosis, and American College of Rheumatology criteria for fibromyalgia diagnosis. Factors such as cognitive failure, depression, anxiety, and sleep disturbances were assessed by patient reported questionnaires. Clinical factors such as BVAS, vasculitis damage index, CRP and BMI were also collected.

**Findings:**

Our AAV cohort comprised 52 patients, with a mean age of 44.7 (20–79), 57% (30/52) of the patients were female. We found 51.9% (27/52) of patients fulfilled the diagnostic criteria for ME/CFS, with 37% (10/27) of those having comorbid fibromyalgia. Rates of fatigue were higher in MPO-ANCA patients, than in PR3-ANCA patients, and their symptoms were more similar to the fibromyalgia controls. Fatigue in PR3-ANCA patients was related to inflammatory markers. These differences may be due to the varied pathophysiology of the PR3- and MPO-ANCA serotypes.

**Interpretation:**

A large proportion of AAV patients suffer from debilitating fatigue consequential enough to meet the diagnostic criteria for ME/CFS. Fatigue associations were not the same between PR3- and MPO-ANCA patients, suggesting that the underlying mechanisms may be different. Future studies should consider ANCA serotype, as further research may inform different clinical treatment strategies for AAV patients suffering from ME/CFS.

**Funding:**

This manuscript was funded by the 10.13039/501100002997Dutch Kidney Foundation (17PhD01).

**Research in context**

**Evidence before this study**

Persistent fatigue plaques many individuals with systemic autoimmune rheumatic diseases, although little is known about its properties in patients with vasculitis. We recently conducted what we believe is the first literature review of studies investigating fatigue in vasculitis patients and found that although most studies found fatigue to be a common and significant factor for vasculitis patients, the data were difficult to compare due to variability in patient populations with regards to vasculitis type and the use of multiple fatigue scoring tools. What we uncovered was that fatigue in vasculitis patients was less likely to be associated with disease factors such as disease subtype, disease activity, disease duration and organ involvement. Instead, fatigue was more commonly associated with pain, anxiety, depression and quality of life, with some studies also indicating a possible association with sleep disturbances and impaired cognitive function.

Previous studies investigating fatigue in anti-neutrophil cytoplasmic antibody associated vasculitis (AAV) patients, separated patients based on vasculitis subtype (granulomatosis with polyangiitis (GPA), microscopic polyangiitis (MPA), eosinophilic GPA (EGPA)), and the findings regarding differences in fatigue between these groups were inconclusive. We and others have suggested that the distinction between ANCA serotype is more important than between vasculitis subtypes. Many clinical and genetic differences exist between patients with PR3-AAV and MPO-AAV, suggesting separation of patients by serotype may indeed be more informative in a research setting.

**Added value of this study**

Our study is to our knowledge, the first looking at the prevalence of clinically defined ME/CFS and fibromyalgia in AAV patients. We found that 54% of AAV patients fulfilled the diagnostic criteria for ME/CFS. Rates of fatigue and fatigue associated symptoms differed between PR3-AAV and MPO-AAV patients, a finding which is supported by the varied pathophysiology of the PR3- and MPO-ANCA serotypes.

**Implications of all the available evidence**

Fatigue in AAV patients is severe and meets the diagnostic criteria of ME/CFS, and the clinical features may differ by serotype—suggesting potential mechanistic differences related to fatigue between MPO-AAV and PR3-AAV. Our findings may result in future studies exploring these differences which may result in improved treatment strategies for patients with AAV and other systemic rheumatic diseases (in general).

## Introduction

Persistent fatigue is frequently associated with systemic autoimmune rheumatic diseases (SARDs),^1^, ^2^, ^3^ and has a profound impact on patient's quality of life. Studies investigating fatigue in SARDs however, lack consistency, with investigators using various different tools to define fatigue, in markedly varied patient groups.^3^ This has resulted in poor clinical management of fatigue in these patients, with patients often feeling dismissed by their physicians.^4^ Though many studies have investigated fatigue in Rheumatoid arthritis (RA),^2^^,^^5^ Systemic lupus erythematosus (SLE),^5^ and systemic sclerosis (SSc),^1^ much less has been documented about fatigue in primary systemic vasculitis (PSV), a concerning finding since the prevalence of fatigue in PSV is high (up to 91.2%).^3^

In PSV, fatigue has been shown to be associated with factors such as pain, poor sleep quality, anxiety and depression, and less so with disease specific factors such as disease activity/severity or organ involvement.^3^ Treatment of patients with immunosuppressive drugs has only shown mild improvement in fatigue levels in some patients, with no improvement in others.^6^^,^^7^ The symptoms associated with fatigue in PSV, are similar to the symptoms experienced by patients with either myalgic encephalomyelitis/chronic fatigue syndrome (ME/CFS)^8^ or fibromyalgia.^9^ Though not identical, a number of similarities are present in various pathways which result in immune and metabolic dysregulation in PSV and ME/CFS.^3^

Primary systemic vasculitis (PSV) is an umbrella term, used to cover many different disorders characterized by inflammation of the blood vessels without a known underlying infectious etiology. PSV are often classified based on the size of the vessels involved. Small-vessel vasculitides (SVV) include anti-neutrophil cytoplasmic antibody (ANCA)-associated vasculitis (AAV)^10^ and can be subclassified further into granulomatosis with polyangiitis (GPA), microscopic polyangiitis (MPA), eosinophilic GPA (EGPA) and renal-limited vasculitis.^10^

AAV patients have antibodies directed against proteinase 3 (PR3) or myeloperoxidase (MPO). PR3-ANCA is most commonly associated with GPA, and MPO-ANCA with MPA and EGPA, but these associations are not exclusive.^11^^,^^12^ Notably, a substantial proportion of EGPA patients, and to a lesser extent GPA and MPA patients, are ANCA negative.^13^ Thus, the distinction between GPA and MPA in the context of ANCA serotype is flawed and highlights the discordance between disease characterization and ANCA serotype categorization.^14^ Many clinical and genetic differences exist between patients with PR3-AAV and MPO-AAV, including age of onset, organ involvement, and single-nucleotide polymorphisms (SNPs) in the Human Leucocyte Antigen (HLA). Differences in rates of relapse and patient survival are also evident.^13^^,^^14^ The majority of studies looking into the role of fatigue in vasculitis have focused on disease subtype, and though some have investigated fatigue in AAV, few have compared differences between the PR3-ANCA and MPO-ANCA serotypes.^3^

As a result, we aimed to identify whether AAV patients fulfil the diagnostic criteria for ME/CFS and fibromyalgia in this study. In addition, we sought to determine whether differences in fatigue correlate between PR3-ANCA and MPO-ANCA patients. We hypothesize that a large proportion of AAV patients will fulfil the diagnostic criteria for both ME/CFS and fibromyalgia and that differences will be found between PR3 and MPO patients.

## Methods

### Patients and controls

Study participants were referred to the rheumatology department of the University of Alberta Hospital between January 2019 and April 2022. Accompanying healthy individuals were asked to take part as controls. Written informed consent was obtaining for all participants. The University of Alberta Research Ethics Board (Pro00085583, Pro00090050) approved the study. Patients with a positive PR3-ANCA or a positive MPO-ANCA and a diagnosis of AAV were included in our study. AAV diagnosis was based on previously described criteria.^15^ PR3-ANCA and MPO-ANCA subtype were detected according to the 2017 International consensus for ANCA testing^16^ (Alberta Precision Labs, Edmonton). Exclusion criteria included: active disease, only patients with a Birmingham Vasculitis Activity Score (BVAS) score of 0 were included. For both patients and controls, exclusion criteria included cancer, diabetes and thyroid dysfunction.

Clinical parameters such as BMI, C-reactive protein (CRP) and thyroid stimulating hormone (TSH) levels, were also collected. Organ involvement was based on previously described methods.^11^ Diagnosis of ME/CFS was based on the Canadian Consensus Criteria (2003).^17^^,^^18^ Using the DePaul symptom questionnaire,^19^ patients who meet five or more of the six criteria are considered to have ME/CFS (Table 1). Fibromyalgia was diagnosed, using the modified 2010 American College of Rheumatology criteria for fibromyalgia.^22^^,^^23^ All participants completed validated questionnaires evaluating fatigue, mental and psychological comorbidities sleep disturbances, pain, disease activity, and chronic vasculitis damage.

**Table 1:** Questionnaires for patient symptom assessment.

Questionnaire	Abbreviation	Summary	Reference
The DePaul symptom questionnaire	DSQ	To determine frequency and severity of ME/CFS symptoms. Utilized in the Canadian consensus criteria; where patients who meet ≥5 of the six criteria are considered to have ME/CFS.^17^^,^^18^	^19^
Short Form (36) health survey	SF-36	Lower scores indicate higher levels of fatigue and disability (Scale 0–100).	^20^
Multidimensional fatigue inventory	MFI	20-item scale evaluating five dimensions of fatigue. Higher scores represent higher levels of fatigue.	^21^
The American College of Rheumatology preliminary diagnostic criteria for fibromyalgia and measurement of symptoms severity	WPI SSS	To evaluate widespread pain index (WPI) and symptoms severity of score (SSS). To fulfil the diagnosis of fibromyalgia: the WPI score is greater than or equal to 7 AND the SSS score is greater than or equal to 5 or the WPI score is from 3 to 6 AND the SSS score is greater than or equal to 9.	^22^^,^^23^
The Hospital anxiety and depression scale	HADS	14-item self-reported screening scale. Scores of ≥11 indicate abnormal results.	^24^
Pittsburgh sleep quality index	PSQI	PSQI score has a range of 0–21. A score of ≥5 indicates poorer sleep quality.	^25^
Cognitive failure questionnaire	CFQ	Score range is 0–100. Scores of ≥43 indicate cognitive impairment.	^26^
Birmingham vasculitis activity score-version 3	BVAS	Measure of current vasculitis disease activity. Score range 0–63. Higher scores indicate greater disease activity.	^27^
Vasculitis damage index	VDI	Records presence of any organ damage since the diagnosis. 64 items of damage (grouped into 11 organ-based systems). Higher scores indicate more damage.	^28^

### Questionnaires

All the validated questionnaires used to evaluate patient symptoms of fatigue, fibromyalgia and vasculitis are listed in Table 1.^17^, ^18^, ^19^, ^20^, ^21^, ^22^, ^23^, ^24^, ^25^, ^26^, ^27^, ^28^

### Statistical analysis

All statistical analysis, including; Chi Square for the analysis of qualitative variables, Wilcoxon Rank Sum tests for continuous variables, as well as pairwise correlations with Bonferroni correction were completed using STATA 17.^29^ Missing data frequencies were low and as such missing data was dropped.

### Role of the funding source

This manuscript was funded by the Dutch Kidney Foundation (17PhD01). The study funders did not have any involvement in the study design; in the collection, analysis, and interpretation of data; in the writing of the report; or in the decision to submit the paper for publication.

## Results

### Demographics and clinical characteristics of the study population

Our AAV cohort included 60 consecutive patients, 52 of which were selected for inclusion into this study. The 8 patients excluded had active disease (BVAS > 0). Thirty healthy controls (HC) and 30 fibromyalgia patients (FM) were also sequentially included in our study as controls.

AAV, FM, and HC groups were similar with regards to marital status, education, and BMI (Table 2). Significant differences were observed for employment status (p = 0.004), sex (p ≤ 0.001), age (p = 0.02), and CRP (p < 0.001) (Table 2). These observations reflect the more advanced age of AAV patients, many of whom were retired, and a female dominance in fibromyalgia. CRP values were highest in the AAV patient group (Table 2). Twenty-seven (51.9%) of the 52 AAV patients met the diagnostic criteria for ME/CFS, of those, 10/27 (37%) met the criteria for FM (Table 2). All 30 (100%) FM patients met the criteria for both FM and ME/CFS; none of the healthy controls met either of these criteria. Vasculitis subtype was not associated with fatigue (p = 0.59), but a trend was evident for serotype, with 59.2% (16/27) of AAV-CFS patients being MPO-ANCA positive, compared to 40.7% (11/27) PR3-ANCA positive (p = 0.08) (Table 3). VDI scores were significantly higher in AAV-CFS patients (p = 0.02). Organ involvement between both groups was similar, though a trend was observed for increased Ear, Nose and Throat (ENT) (p = 0.05), and significantly more eye involvement (p = 0.03) in AAV-CFS patients (Table 3). Medication history did not differ between AAV-CFS and AAV-NCFS patients (Table 3).

**Table 2:** Patient and control participant characteristics.

Categorical variable	Healthy (n = 30)	Vasculitis (n = 52)	Fibromyalgia (n = 30)	p-value
	Number (percentage)	Number (percentage)	Number (percentage)	
Sex (F)	20/30 (66.7)	30/52 (57.6)	30/30 (100)	**<0.001**
Marital status				
*Single*	4/29 (13.7)	9/50 (18.0)	0/30 (0.0)	
*Married*	23/29 (79.3)	33/50 (66.0)	25/30 (83.3)	
*Separated/divorced*	2/29 (6.8)	4/50 (8.0)	5/30 (16.6)	
*Widowed*	0/29 (0.0)	4/50 (8.0)	0/30 (0.0)	0.05
Education				
*<High school*	3/28 (10.7)	2/47 (4.2)	0/26 (0.0)	
*High school*	5/28 (17.8)	15/47 (31.9)	7/26 (26.9)	
*Graduate degree*	13/28 (46.4)	20/47 (42.5)	15/26 (57.6)	
*Professional degree*	7/28 (25.0)	10/47 (21.2)	4/26 (15.3)	0.43
Employment				
*Disability*	0/28 (0.0)	5/50 (10.0)	1/29 (3.4)	
*Student*	2/28 (7.1)	1/50 (2.0)	0/29 (0.0)	
*Homemaker*	1/28 (3.5)	0/50 (0.0)	2/29 (6.8)	
*Retired*	1/28 (3.5)	17/50 (34.0)	2/29 (6.8)	
*Unemployed*	2/28 (7.1)	1/50 (2.0)	3/29 (10.3)	
*Employed*	22/28 (78.5)	26/50 (52.0)	21/29 (72.4)	**0.004**
ME/CFS				
*No*^a^	24/30 (80.0)	18/52 (34.6)	0/30 (0.0)	
*Atypical*^b^	6/30 (20.0)	7/52 (13.4)	0/30 (0.0)	
*Yes*^c^	0/30 (0.0)	27/52 (51.9)	30/30 (100)	**<0.001**
Fibromyalgia (Y)	0/30 (0.0)	10/52 (19.2)	30/30 (100)	**<0.001**
**Continuous variables**	**Mean (SD)**	**Mean (SD)**	**Mean (SD)**	

Age (yrs)	44.7 (12.6)	53.3 (15.8)	44.3 (9.9)	**0.02**
BMI (kg/m^2^)	27.4 (6.2)	29.0 (5.6)	26.6 (6.9)	0.30
CRP	3.4 (4.3)	6.3 (10.2)	4.2 (4.6)	**<0.001**

Significant p values are indicated in bold.

Y-Yes; F-Female; ME/CFS-Myalgic encephalomyelitis/chronic fatigue syndrome; BMI-Body mass index; CRP-C reactive protein.

a No fatigue, defined as 0–2 criteria based on the Canadian consensus criteria.

b Atypical, defined as 3–4 criteria based on the Canadian consensus criteria.

c ME/CFS, defined as 5–6 criteria based on the Canadian consensus criteria.

**Table 3:** Comparison of fatigue in AAV subtype and serotype.

Categorical variable	AAV-NCFS (n = 18)	AAV-CFS (n = 27)	p-value
	Number (percentage)	Number (percentage)	
Subtype			
GPA	10/18 (55.5)	19/27 (70.3)	
MPA	4/18 (22.2)	4/27 (14.8)	
EGPA	4/18 (22.2)	4/27 (14.8)	0.59
Serotype			
PR3-ANCA	12/18 (66.6)	11/27 (40.7)	
MPO-ANCA	6/18 (33.3)	16/27 (59.2)	0.08
*Organ involvement*			
Ear, Nose, Throat (ENT)	14/18 (77.7)	26/27 (96.2)	0.05
Lung	12/18 (66.6)	14/27 (51.8)	0.32
Kidney	11/18 (61.1)	13/27 (48.1)	0.39
Skin	9/18 (50.0)	11/27 (40.7)	0.54
Nerves	6/18 (33.3)	9/27 (33.3)	1.0
Eye	3/18 (16.6)	13/27 (48.1)	**0.03**
Gastrointestinal involvement	3/18 (16.6)	2/27 (7.4)	0.33
Arthralgia	11/18 (61.1)	22/27 (81.4)	0.13
Pathology (positive)	13/18 (72.2)	18/27 (66.6)	0.69
*Medication history*			
Prednisone	16/18 (88.8)	21/27 (77.7)	0.34
Cyclophosphamide	10/18 (55.5)	15/27 (55.5)	1.0
Methotrexate	6/18 (33.3)	10/27 (37.0)	0.79
Azathioprine	10/18 (55.5)	11/27 (40.7)	0.32
Rituximab	8/18 (44.4)	10/27 (37.0)	0.61
Cellcept	1/18 (5.5)	0/27 (0.0)	0.21
Mepolizumab	0/18 (0.0)	2/27 (7.4)	0.23
**Continuous variables**	**Median (IQR)**	**Median (IQR)**	

VDI	1.5 (1; 2)	2 (1; 3)	**0.02**
Duration	2 (1; 5)	1 (1; 6)	0.97

AAV patients n = 45, due to the exclusion of nine atypically fatigue patients as defined by 3 and 4 positive criteria, based on the Canadian consensus criteria.

Significant p values are indicated in bold.

AAV-NCFS-ANCA vasculitis patients without ME/CFS; AAV-CFS-ANCA vasculitis patients with ME/CFS; GPA- Granulomatosis with polyangiitis; MPA- Microscopic polyangiitis, EGPA- Eosinophilic granulomatosis with polyangiitis.

Though disease subtype was mixed between PR3- and MPO-ANCA patients, GPA was most common in both groups (84.6% vs 50%), and MPA higher in the MPO-ANCA patients (30.7% vs 3.8%) (p = 0.01) (Supplementary Table S1). At the time of inclusion, all patients had inactive disease, with BVAS scores of zero. BMI (28.0 vs 28.4), CRP levels (3.45 vs 3.35 mg/L), as well as disease duration (4 vs 1 years) and VDI, were similar between MPO-ANCA and PR3-ANCA patients (Supplementary Table S1). Organ involvement was similar between both groups with the exception of kidney involvement which was more prevalent in PR3-ANCA patients (p = 0.02). Arthralgia (p = 0.03) and a positive pathology finding (p = 0.02) were also more prominent in PR3-ANCA patients. Anxiety was more prevalent in MPO-ANCA patients (p = 0.01) (Supplementary Table S1).

### Fatigue and disease related measures in fatigued versus non-fatigued AAV patients

For the comparison of fatigue related measures, patients with atypical fatigue were excluded; as these patients, did not fulfil the classification criteria for ME/CFS and were also not non-fatigued. In total, forty-five AAV patients were included; 18 without fatigue (AAV-NCFS) and 27 fulfilling ME/CFS criteria (AAV-CFS).

Comparing non-fatigued AAV patients (AAV-NCFS) (n = 18) to FM patients, we found that AAV-NCFS patients and healthy controls were statistically different from FM patients with respect to cognitive failure, anxiety, depression and sleep disturbances (p < 0.05) (Table 4). AAV-NCFS patients and healthy controls also had significantly reduced levels of pain (as evident in the WPI score), as well as significantly lower fatigue scores as measured by the symptom severity score (SSS), and multidimensional fatigue inventory (MFI) when compared to the FM controls (p < 0.05). Both AAV-NCFS patients and healthy controls had better quality of life (SF-36) than the FM controls (p < 0.05). The only difference between AAV-NCFS patients and healthy controls was that AAV-NCFS patients were significantly older than the FM controls (p = 0.007) (Table 4).

**Table 4:** Comparison of fatigue related variables in healthy controls, AAV-NCFS and AAV-CFS as compared to fibromyalgia patients.

Categorical variables	Fibro (n = 30)	Healthy (n = 30)	p-value	AAV-NCFS (n = 18)	p-value	AAV-CFS (n = 27)	p-value
	Number (%)	Number (%)		Number (%)		Number (%)	
Fibromyalgia (Y)	30/30 (100)	0/30 (0.0)	**<0.001**	0/18 (0.0)	**<0.001**	10/27 (37.0)	**<0.001**
Cognitive failure (Y)	23/28 (82.1)	4/30 (13.3)	**<0.001**	1/18 (5.5)	**<0.001**	16/27 (59.2)	0.06
Anxiety (Y)	16/30 (53.3)	0/30 (0.0)	**<0.001**	0/18 (0.0)	**<0.001**	8/27 (29.6)	0.07
Depression (Y)	14/30 (46.6)	1/30 (3.3)	**<0.001**	0/18 (0.0)	**0.001**	4/27 (14.8)	**0.01**
Sleep disturbances (Y)	29/30 (96.6)	15/30 (50.0)	**<0.001**	14/18 (77.7)	**0.03**	21/26 (80.7)	0.05
Sex (F)	30/30 (100)	20/30 (66.6)	**0.001**	3/18 (16.6)	**<0.001**	21/27 (77.7)	**0.006**
**Continuous variables**	**Median (IQR)**	**Median (IQR)**		**Median (IQR)**		**Median (IQR)**	

WPI	10.5 (9; 16)	1.5 (0; 2)	**<0.001**	1.5 (0; 2.5)	**<0.001**	4.5 (1.5; 7)	**<0.001**
SSS	9 (8; 11)	2 (1; 3)	**<0.001**	3 (1; 4)	**<0.001**	6.5 (5; 8.5)	**<0.001**
MFI	75 (65; 88)	35 (29; 47)	**<0.001**	44.5 (38; 51)	**<0.001**	70 (62; 81)	0.17
SF-36	33.2 (26.3; 46.7)	86.4 (76.5; 94.7)	**<0.001**	70.6 (60.0; 80.6)	**<0.001**	49.5 (33.1; 61.8)	**0.02**
BMI	25.1 (21; 32.1)	27.3 (21.6; 30.4)	0.44	27.9 (25.3; 30.1)	0.14	27.2 (24.8; 31.2)	0.10
CRP	2.6 (0.6; 5)	1.9 (0.5; 5.2)	0.58	2.8 (0.8; 4.8)	0.66	4.1 (1.4; 5.7)	0.20
Age	42.5 (39; 51)	47.5 (35; 56)	0.78	54 (45; 65)	**0.007**	57 (46; 63)	**<0.001**

AAV patients n = 45, due to the exclusion of nine atypically fatigue patients as defined by the DSQ-2.

Significant p values are indicated in bold.

AAV-NCFS-ANCA vasculitis patients without ME/CFS; AAV-CFS-ANCA vasculitis patients with ME/CFS; Y-Yes; F-Female; BMI-Body mass index; CRP-C reactive protein; WPI-Widespread pain index; SSS-Symptom severity score; SF36-Short form 36; MFI-Multidimensional fatigue inventory.

In contrast, in AAV-CFS patients (n = 27) similar rates of cognitive failure (59.2% (16/27) vs 82.1% (23/28)) (p = 0.06), anxiety (29.6% (8/27) vs 53.3% (16/30)) (p = 0.07) and sleep disturbance (80.7% (21/26) vs 96.6% (29/30)) (p = 0.05), when compared to FM patients. Fatigue as measured by the MFI was similar between AAV-CFS patients (score = 70) and FM patients (score = 75) (p = 0.17), as were BMI (27.2 vs 25.1) (p = 0.10), and CRP levels (4.1 vs 2.6) (p = 0.20) (Table 4).

### Fatigue: PR3-ANCA versus MPO-ANCA patients

Rates of ME/CFS were higher in MPO-ANCA patients than in PR3-ANCA patients, with 61.5% (16/26) of MPO-ANCA patients having ME/CFS, compared to 42.3% (11/26) of PR3-ANCA patients, though this was not statistically significant (Supplementary Table S1). Differences were evident when comparing non-fatigued and fatigued patients within the two serotypes. Fatigued PR3-ANCA patients had higher rates of cognitive failure (p = 0.001) and arthralgia (p = 0.03), more females (<0.001), and higher levels of CRP (p = 0.03), with a trend also evident for fibromyalgia symptoms (p = 0.05) (Supplementary Table S2). In MPO-ANCA patients, fatigue was associated with pain as measured by the WPI (p = 0.01), with trends evident for fibromyalgia (p = 0.05) and anxiety (p = 0.05). Organ involvement, medication history and VDI were not different in fatigued in PR3-ANCA or MPO-ANCA patients (p < 0.05), compared to non-fatigued patients. Fatigued PR3- and MPO-ANCA patients experienced reduced levels in quality of life as noted by the SSS, MFI, and SF36 (p < 0.05) (Supplementary Table S2).

Separating fatigued AAV patients by serotype (PR3-ANCA n = 11; MPO-ANCA n = 16), and comparing them to fibromyalgia controls, revealed several notable differences between the two groups. PR3-AAV-CFS and MPO-AAV-CFS patients were less often diagnosed with FM (p ≤ 0.001), but had comparable rates of cognitive failure (Table 5). Intriguingly, anxiety, depression and sleep disturbances were less common in PR3-AAV-CFS patients (p = 0.01; p = 0.02; p = 0.01), compared to FM controls. MPO-AAV-CFS patients: however, had similar frequencies of anxiety, depression and sleep disturbance diagnoses as FM patients (Table 5).

**Table 5:** Comparison of demographic, disease and fatigue related variables in fatigued PR3-ANCA and MPO-ANCA patients.

Categorical variables	Fibromyalgia (n = 30)	PR3-AAV-CFS (n = 11)	p-value	MPO-AAV-CFS (n = 16)	p-value
		Number (%)		Number (%)	
Fibromyalgia (Y)	30/30 (100)	3/11 (27.3)	**<0.001**	7/16 (43.7)	**<0.001**
Cognitive failure (Y)	23/28 (82.1)	7/11 (63.6)	0.21	9/16 (56.2)	0.06
Anxiety (Y)	16/30 (53.3)	1/11 (9.0)	**0.01**	7/16 (43.7)	0.53
Depression (Y)	14/30 (46.6)	1/11 (9.0)	**0.02**	3/16 (18.7)	0.06
Sleep Disturb.(Y)	29/30 (96.6)	7/10 (70.0)	**0.01**	14/16 (87.5)	0.23
Sex (F)	30/30 (100)	9/11 (81.8)	**0.01**	12/16 (75.0)	**0.004**
Marital status					
*Single*	0/30 (0.0)	1/11 (9.0)		1/15 (6.7)	
*Married*	25/30 (83.3)	7/11 (63.6)		10/15 (66.7)	
*Separated/divorced*	5/30 (16.6)	1/11 (9.0)		3/15 (20.0)	
*Widowed*	0/30 (0.0)	2/11 (18.2)	**0.03**	1/15 (6.7)	0.22
Education					
*<High school*	0/26 (0.0)	0/10 (0.0)		2/13 (15.3)	
*High school/partial*	7/26 (26.9)	5/10 (50.0)		2/13 (15.3)	
*Graduate degree*	15/26 (57.6)	4/10 (40.0)		6/13 (46.1)	
*Professional degree*	4/26 (15.3)	1/10 (10.0)	0.42	3/13 (23.0)	0.17
Employment					
*Disability*	1/29 (3.4)	3/10 (30.0)		2/16 (12.5)	
*Homemaker*	2/29 (6.8)	0/10 (0.0)		0/16 (0.0)	
*Retired*	2/29 (6.8)	2/10 (20.0)		7/16 (43.7)	
*Unemployed*	3/29 (10.3)	0/10 (0.0)		0/16 (0.0)	
*Employed*	21/29 (72.4)	5/10 (50.0)	0.07	7/16 (43.7)	**0.01**
**Continuous variables**	**Median (IQR)**	**Median (IQR)**		**Median (IQR)**	

WPI	10.5 (9; 16)	4 (1; 7)	**<0.001**	5.5 (2.5; 7)	**<0.001**
SSS	9 (8; 11)	6 (4; 7)	**<0.001**	6.0 (5; 8)	**0.002**
MFI	75 (65; 88)	70 (62; 71)	0.20	66 (61; 81)	0.32
SF-36	33.2 (26.3; 46.7)	52.1 (33.1; 63)	**0.02**	49.5 (31.1; 56.2)	0.10
Age (yrs)	42.5 (39; 51)	53 (40; 58)	0.16	62.5 (55.5; 69.5)	**<0.001**
BMI (kg/m^2^)	25.1 (21; 32.1)	28.7 (23; 38.4)	0.10	27 (24.8; 31.2)	0.29
CRP	2.55 (0.6; 5)	5.3 (3.7; 24.7)	0.11	3.35 (1.25; 5.7)	0.55
Sleep Disturb. score	13 (11; 16)	6.5 (4; 13)	**0.01**	10.5 (6.5; 16.5)	0.14
Anxiety score	11 (8; 14)	5 (5; 10)	**0.001**	7.5 (3.5; 12)	**0.04**
Depression score	9.5 (5; 13)	6 (3; 8)	0.07	5.5 (5; 9)	**0.03**
Cognitive fail. score	58.5 (47.5; 71)	43 (21; 46)	**0.001**	48.5 (31; 65.5)	0.10

Significant p values are indicated in bold.

Y-Yes; F-Female; BMI-Body mass index; CRP-C reactive protein; WPI-Widespread pain index; SSS-Symptom severity score; SF36-Short form 36; MFI-Multidimensional fatigue inventory.

**Fig. 1:**
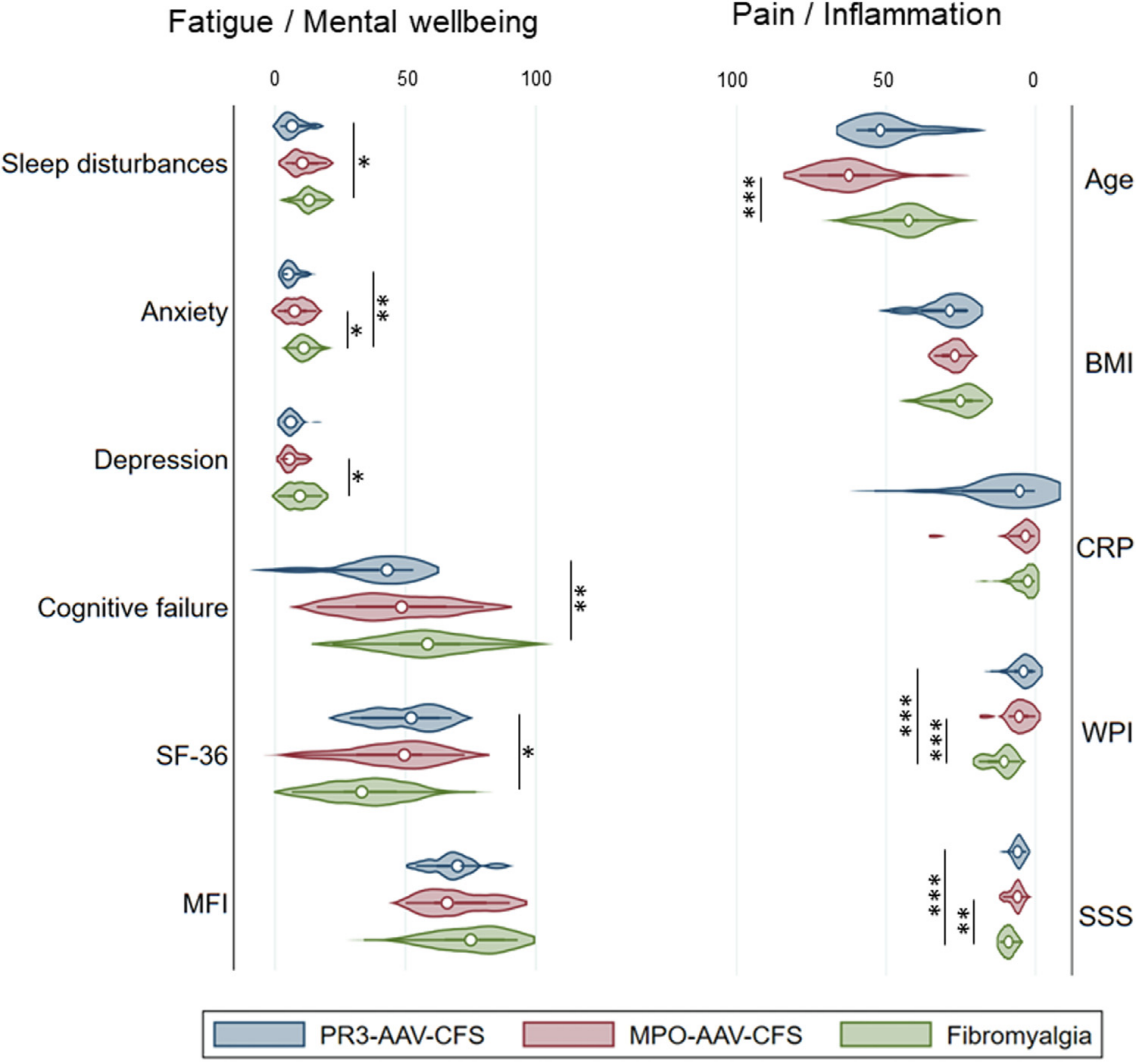
**Comparison of fatigue/mental wellbeing and pain/inflammation between fatigued PR3- and MPO-ANCA patients and fibromyalgia controls.** Violin plots showing median and interquartile range. ∗p < 0.05, ∗∗p < 0.01, ∗∗∗p < 0.001.

Pain as measured by the WPI, was lower in PR3- and MPO-AAV-CFS patients than in fibromyalgia controls (p < 0.001) (Table 5). The localization of pain reported by patients is shown in Supplementary Fig. S1. As expected, the vast majority of FM control patients had pain in all areas. PR3-AAV-CFS patient complaints were mostly of neck, shoulder and upper back pain, whereas, in addition to these, MPO-AAV-CFS patients also reported high levels of lower back, abdominal and lower leg pain (Supplementary Fig. S1). Fatigue as measured by the MFI, as well as BMI and CRP, were similar between all three groups (p > 0.05), but quality of life as measured by the SF36 was better in PR3-AAV-CFS patients than in FM controls (p = 0.02) (Table 5, Fig. 1). There was no significant difference between the five dimensions of the MFI, or the eight scales of the SF36 between PR3- and MPO-AAV-CFS patients (data not shown). MPO-AAV-CFS patients were older (p < 0.001) (Table 5, Fig. 1).

### Correlates of fatigue in PR3-ANCA and MPO-ANCA patients

**Fig. 2:**
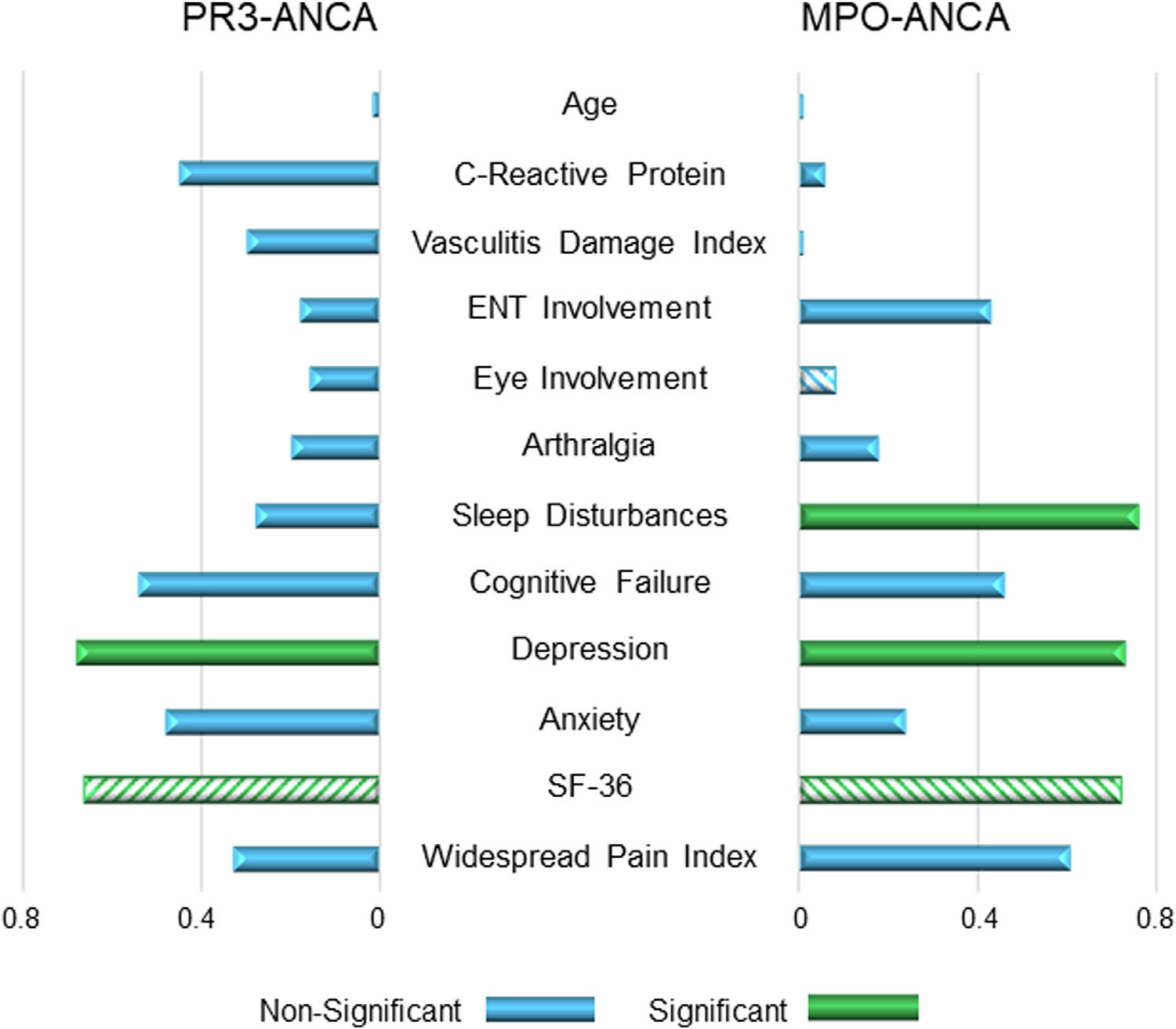
**Pairwise correlations with multidimensional fatigue inventory.** Comparison of fatigue correlates between PR3- and MPO-ANCA patients. Diagonal stripes indicate negative correlations.

We analyzed the correlates of fatigue as measured by the MFI, for both PR3-ANCA and MPO-ANCA patients. Depression (p = 0.01; p = 0.003) and quality of life (SF-36) (p = 0.02; p = 0.004) were significantly correlated with fatigue in both groups (Fig. 2, Supplementary Tables S3 and S4). In MPO-ANCA, fatigue was also correlated with Sleep disturbances (p = 0.001) (Fig. 2, Supplementary Table S4). No correlates were evident for disease related factors such as VDI, organ involvement or medication history.

## Discussion

We believe this to be the first manuscript investigating the presence of both ME/CFS and fibromyalgia in AAV patients. All our AAV patients had inactive disease (BVAS = 0), yet we found that over half of fulfil the diagnostic criteria for ME/CFS, and that a third of those patients suffered from fibromyalgia.

These debilitating symptoms, seemingly unassociated with disease activity itself, requires desperate attention. A recent study by Monti et al.,^30^ found that 40% of AAV patients classified as being in clinical disease remission, score themselves as having active disease, based on the Patient Global Assessment scale. They found fatigue and pain to be the strongest correlates of patient global assessment (PtGA).^30^

Vasculitis subtype had no bearing on fatigue, but the MPO-ANCA serotype displayed appreciably higher rates of fatigue. This may reflect the more chronic, smoldering course of MPO-ANCA disease, compared to the acute but relapsing nature of PR3-ANCA.^14^^,^^31^^,^^32^ This finding is further supported by a study done by Basu et al.,^33^ where, in the supplementary data, it was shown that patients who are MPO-ANCA positive, were more likely to have higher rates of fatigue than MPO-ANCA negative patients (p = 0.05). In contrast, patients who were currently PR3-ANCA positive did not have higher rates of fatigue than PR3-ANCA negative patients did (p = 0.47).^33^

When we compared PR3- and MPO-ANCA fatigued patients, MPO-AAV-CFS patients had more similarities with fibromyalgia patients than PR3-AAV-CFS. MPO-AAV-CFS patients also had higher levels of pain than PR3-AAV-CFS patients, and higher rates of comorbid fibromyalgia. The finding that MPO-AAV-CFS patients have higher levels of lower leg pain than PR3-AAV-CFS patients is also of interest. Lower leg pain has shown to be associated with sympathetic dysfunction particularly in patients with postural tachycardia syndrome (POTS)^34^ - a common feature in ME/CFS. A recent paper also found that the most frequent pain complaint in AAV patients was leg pain.^30^ PR3-AAV-CFS patients showed little similarities with FM patients. PR3-AAV-CFS fatigue was associated with a female sex, and having a higher CRP. The higher CRP levels in PR3-AAV may reflect ongoing rhinosinusitis, which is a common cause of low-grade inflammation in these patients.^35^ The unexpected finding that arthralgia is more prevalent in non-fatigued PR3-ANCA patients, is intriguing, and warrants further investigation.

Our study has several limitations. First, as there are currently no objective biomarkers for fatigue or for ME/CFS; this study is limited to diagnosis based on validated patient reported questionnaires. Additionally, our single center design and relatively small sample size will require future larger more ethnically diverse multi-centered cohorts to confirm our results. The FM control group was younger and all female, compared to the AAV group and we cannot exclude the possibility that this may have affected our results. Lastly, we were unable to report disaggregated sex data as our small cohort was prohibitive. Gender and ethnicity data were not collected.

Fatigue in AAV patients is severe and meets the diagnostic criteria of ME/CFS. We emphasize that physicians should be aware of this when assessing treatment and management strategies for improving quality of life in AAV patients. Furthermore, although mechanistically unexplained, the underlying mechanisms behind patient-reported fatigue may differ by serotype in AAV. More research is required to help elucidate these distinctions in the future. Understanding the differences behind the development of fatigue between the two serotypes, will not only help inform future clinical trials and treatment strategies in AAV, but may possibly also inform fatigue mechanisms in other systemic rheumatic diseases, and potentially lead to the development of serotype specific biomarkers that can be utilized in clinical trials for these patients.

## Contributors

Conceptualization: C.v.E., M.S.O., J.W.C.T.; Data curation: D.R., N.M., E.Y., A.C., A.S.R.; Formal Analysis: C.v.E.; Funding Acquisition: J.W.C.T.; Project administration: D.R., N.M.; Supervision: M.S.O., J.W.C.T.; Writing—original draft: C.v.E.; Writing review and editing: C.v.E., E.Y., A.C., A.S.R, M.S.O, J.W.C.T.; Data verification: C.v.E., D.R., J.W.C.T.; Access to raw data: J.W.C.T., D.R.; Responsible for study: J.W.C.T.

## Data sharing statement

Data collected for this study, including individual participant data, will not be made available to others.

## Declaration of interests

This manuscript was funded by the Dutch Kidney Foundation (17PhD01). JWCT and MO's research is supported by unrestricted grants from the 10.13039/501100000190University of Alberta (Canada), 10.13039/501100002997Dutch Kidney Foundation, the 10.13039/501100018911University Hospital Foundation and Kaye Grants, Scleroderma Canada, and 10.13039/100001003Boehringer Ingelheim. Additionally, MO is funded by the 10.13039/501100000142Arthritis Society (STAR/IMHA career development award 00049) and Scleroderma Canada. JWCT received speaker honoraria Pfizer and Medexus. JWCT on IDMC of InflaRx until 2021, Chair of IDMC for complement inhibitor. MO received speaker honoraria from Boehringer Ingelheim.
