# Supplementary material for: Myalgic encephalomyelitis/chronic fatigue syndrome (ME/CFS) and fibromyalgia: PR3-versus MPO-ANCA-associated vasculitis, an exploratory cross-sectional study
Source: Lancet Reg Health Am. 2023 Feb 27;20:100460. doi: 10.1016/j.lana.2023.100460 (PMC9986636; doi:10.1016/j.lana.2023.100460)
Supplement: Supplementary Tables S1–S5 and Fig. S1 [file mmc1.docx]

**Supplementary Files**

**Myalgic Encephalomyelitis/Chronic Fatigue Syndrome (ME/CFS) and fibromyalgia: PR3- versus MPO-ANCA-associated vasculitis, an exploratory cross-sectional study**

Charmaine van Eeden, Naima Mohazab, Desiree Redmond, Elaine Yacyshyn, Allison Clifford, Anthony S. Russell, Mohammed S. Osman & Jan Willem Cohen Tervaert

Table of contents:

Supplementary Table 1. Comparison of PR3-ANCA and MPO-ANCA Page 2

Supplementary Table 2. Comparison of demographic, disease and fatigue related variables between

fatigued and non-fatigued PR3- and MPO-ANCA patients Page 3

Supplementary Table 3. Pairwise correlations for PR3-ANCA patients Page 4

Supplementary Table 4. Pairwise correlations for MPO-ANCA patients Page 5

Supplementary Figure 1. Pain distribution in fatigued PR3-ANCA and MPO-ANCA patients, as

compared to fibromyalgia controls Page 6

**Supplementary Table 1. Comparison of PR3-ANCA and MPO-ANCA**

|  | **PR3 (n=26)** | **MPO (n=26)** | **p-value** |
| --- | --- | --- | --- |
| *Categorical variables* | *Number (%)* | *Number (%)* |  |
| ME/CFS  *No*  *Atypical*  *Yes* | 12/26 (46.1)  3/26 (11.5)  11/26 (42.3) | 6/26 (23.0)  4/26 (15.3)  16/26 (61.5) | 0.21 |
| Fibromyalgia (Y) | 3/26 (11.5) | 7/26 (26.9) | 0.15 |
| Cognitive Failure (Y) | 7/26 (26.9) | 11/26 (42.3) | 0.24 |
| Anxiety (Y) | 1/26 (3.8) | 8/26 (30.7) | **0.01** |
| Depression (Y) | 1/26 (3.8) | 3/26 (11.5) | 0.29 |
| Sleep Disturbances (Y) | 19/25 (76.0) | 22/26 (84.6) | 0.43 |
| Gender (F) | 12/26 (46.1) | 18/26 (69.2) | 0.09 |
| Subtype  *GPA*  *MPA*  *EGPA* | 22/26 (84.6)  1/26 (3.8)  3/26 (11.5) | 13/26 (50.0)  8/26 (30.7)  5/26 (19.2) | **0.01** |
| Marital status  *Single*  *Married*  *Separated/Divorced*  *Widowed* | 6/26 (23.0)  17/26 (65.3)  1/26 (3.8)  2/26 (7.6) | 3/24 (12.5)  16/24 (66.6)  3/24 (12.5)  2/24 (8.3) | 0.58 |
| Education  *< High School*  *High School/ Partial degree*  *Graduate degree*  *Professional degree* | 0/25 (0.0)  10/25 (40.0)  11/25 (44.0)  4/25 (16.0) | 2/22 (9.0)  5/22 (22.7)  9/22 (40.9)  6/22 (27.2) | 0.25 |
| Employment  *Disability*  *Student*  *Retired*  *Unemployed*  *Employed* | 3/25 (12.0)  0/25 (0.0)  7/25 (28.0)  1/25 (4.0)  14/25 (56.0) | 2/25 (8.0)  1/25 (4.0)  10/25 (40.0)  0/25 (0.0)  12/25 (48.0) | 0.57 |
| *Organ Involvement*  Ear, Nose, Throat  Lung  Kidney  Skin  Nerves  Eye | 24/26 (92.3)  16/26 (61.5)  18/26 (69.2)  13/26 (50.0)  9/26 (34.6)  6/26 (23.0) | 22/26 (84.6)  14/26 (53.8)  10/26 (38.4)  9/26 (34.6)  8/26 (30.7)  12/26 (46.1) | 0.38  0.57  **0.02**  0.26  0.76  0.08 |
| Gastrointestinal Involvement | 3/26 (11.5) | 2/26 (7.6) | 0.63 |
| Arthralgia | 22/26 (84.6) | 15/26 (57.6) | **0.03** |
| Pathology (positive) | 21/26 (80.7) | 13/26 (50.0) | **0.02** |
| *Medication History*  Prednisone  Cyclophosphamide  Methotrexate  Azathioprine  Rituximab  Cellcept  Mepolizumab | 24/26 (92.3)  16/26 (61.5)  12/26 (46.1)  16/26 (61.5)  13/26 (50.0)  1/26 (3.8)  1/26 (3.8) | 20/26 (76.9)  11/26 (42.3)  6/26 (23.0)  10/26 (38.4)  8/26 (30.7)  0/26 (0.0)  1/26 (3.8) | 0.12  0.16  0.08  0.09  0.15  0.31  1.0 |
| *Continuous variables* | *Median (IQR)* | *Median (IGR)* | *p-value* |
| WPI | 2 (1;4) | 3 (1;6) | 0.32 |
| SSS | 4 (3;6) | 6 (4;7) | **0.04** |
| MFI | 56 (46;65) | 61 (55.5;71.5) | 0.14 |
| SF-36 | 62.4 (46.7;73.8) | 50.6 (43.4;63) | 0.13 |
| Age (yrs) | 52 (40-58) | 61.5 (48;70) | 0.05 |
| BMI (kg/m^2^) | 28.0 (25.3;32.6) | 28.4 (25.5;30.8) | 0.89 |
| VDI | 2 (1;2) | 2 (1;3) | 0.61 |
| CRP | 3.45 (1;5.5) | 3.35 (1.1;5.7) | 0.59 |
| Duration (yrs) | 4 (1;6) | 1 (1;6) | 0.41 |

**Abbreviations:** Y-Yes; F-Female; GPA- Granulomatosis with polyangiitis; MPA- Microscopic polyangiitis, EGPA- Eosinophilic granulomatosis with polyangiitis; BMI-Body mass index; CRP-C reactive protein; WPI-Widespread pain index; SSS-Symptom severity score; SF36-Short form 36; MFI-Multidimensional fatigue inventory; VDI-Vasculitis damage index.

**Supplementary Table 2. Comparison of demographic, disease and fatigue related variables between fatigued and non-fatigued PR3- and MPO-ANCA patients**

|  | **PR3-NCFS (n=12)** | **PR3-CFS (n=11)** | **p-value** | **MPO-NCFS (n=6)** | **MPO-CFS (n=16)** | **p-value** |
| --- | --- | --- | --- | --- | --- | --- |
| *Categorical variables* | *Number (%)* | *Number (%)* |  | *Number (%)* | *Number (%)* |  |
| Fibromyalgia (Y) | 0/12 (0.0) | 3/11 (27.2) | 0.05 | 0/6 (0.0) | 7/16 (43.7) | 0.05 |
| Cognitive Failure (Y) | 0/12 (0.0) | 7/11 (63.6) | **0.001** | 1/6 (16.6) | 9/16 (56.2) | 0.09 |
| Anxiety (Y) | 0/12 (0.0) | 1/11 (9.0) | 0.28 | 0/6 (0.0) | 7/16 (43.7) | 0.05 |
| Depression (Y) | 0/12 (0.0) | 1/11 (9.0) | 0.28 | 0/6 (0.0) | 3/16 (18.7) | 0.25 |
| Sleep Disturbances (Y) | 9/12 (75.0) | 7/10 (70.0) | 0.79 | 5/6 (83.3) | 14/16 (87.5) | 0.80 |
| Gender (F) | 1/12 (8.3) | 9/11 (81.8) | **<0.001** | 2/6 (33.3) | 12/16 (75.0) | 0.07 |
| Subtype  *GPA*  *MPA*  *EGPA* | 9/12 (75)  1/12 (8.3)  2/12 (16.6) | 10/11 (90.9)  0/11 (0.0)  1/11 (9.0) | 0.51 | 1/6 (16.6)  3/6 (50.0)  2/6 (33.3) | 9/16 (56.2)  4/16 (25.0)  3/16 (18.7) | 0.25 |
| Marital status  *Single*  *Married*  *Separated/Divorced*  *Widowed* | 4/12 (33.3)  8/12 (66.6)  0/12 (0.0)  0/12 (0.0) | 1/11 (9.0)  7/11 (63.6)  1/11 (9.0)  2/11 (18.1) | 0.18 | 1/6 (16.6)  4/6 (66.6)  0/6 (0)  1/6 (16.6) | 1/15 (6.6)  10/15 (66.6)  3/15 (20.0)  1/15 (6.6) | 0.55 |
| Education  *< High School*  *High School/Partial*  *Graduate degree*  *Professional degree* | 0/12 (0.0)  3/12 (25.0)  7/12 (58.2)  2/12 (16.6) | 0/10 (0.0)  5/10 (50.0)  4/10 (40.0)  1/10 (10.0) | 0.47 | 0/6 (0.0)  2/6 (33.3)  2/6 (33.3)  2/6 (33.3) | 2/13 (15.3)  2/13 (15.3)  6/13 (46.1)  3/13 (23.0) | 0.59 |
| Employment  *Disability*  *Retired*  *Unemployed*  *Employed* | 0/12 (0.0)  5/12 (41.6)  1/12 (8.3)  6/12 (50.0) | 3/12 (22.2)  2/12 (22.2)  0/12 (0.0)  5/12 (55.5) | 0.15 | 0/6 (0.0)  2/6 (33.3)  0/6 (0.0)  4/6 (66.6) | 2/16 (12.5)  7/16 (43.7)  0/16 (0.0)  7/16 (43.7) | 0.51 |
| *Organ Involvement*  ENT  Lung  Kidney  Skin  Nerves  Eye | 10/12 (83.3)  8/12 (66.6)  9/12 (75.0)  7/12 (58.3)  5/12 (41.6)  1/12 (8.3) | 11/11 (100)  5/11 (45.4)  6/11 (54.5)  5/11 (45.4)  4/11 (36.3)  4/11 (36.3) | 0.15  0.30  0.30  0.53  0.79  0.10 | 4/6 (66.6)  4/6 (66.6)  2/6 (33.3)  2/6 (33.3)  1/6 (16.6)  2/6 (33.3) | 15/16 (93.7)  9/16 (56.2)  7/16 (43.7)  6/16 (37.5)  5/16 (31.2)  9/16 (56.2) | 0.09  0.65  0.65  0.85  0.49  0.33 |
| Gastrointestinal Involvement | 2/12 (16.6) | 1/11 (9.0) | 0.59 | 1/6 (16.6) | 1/16 (6.2) | 0.44 |
| Arthralgia | 8/12 (66.6) | 0/11 (0.0) | **0.03** | 3/6 (50.0) | 11/16 (68.7) | 0.41 |
| Pathology (positive) | 9/12 (75.0) | 9/11 (81.8) | 0.69 | 4/6 (66.6) | 9/16 (56.2) | 0.65 |
| *Medication History*  Prednisone  Cyclophosphamide  Methotrexate  Azathioprine  Rituximab  Cellcept  Mepolizumab | 12/12 (100)  8/12 (66.6)  5/12 (41.5)  8/12 (66.6)  7/12 (58.3)  1/12 (8.3) 0/12 (0.0) | 9/11 (81.8)  6/11 (54.5)  6/11 (54.5)  6/11 (54.5)  4/11 (36.3)  0/11 (0.0)  1/11 (9.0) | 0.12  0.55  0.53  0.55  0.29  0.32  0.28 | 4/6 (66.6)  2/6 (33.3)  1/6 (16.6)  2/6 (33.3)  1/6 (16.6)  0/6 (0.0)  0/6 (0.0) | 12/16 (75.0)  9/16 (56.2)  4/16 (25.0)  5/16 (31.2)  6/16 (37.5)  0/16 (0.0)  1/16 (6.2) | 0.69  0.33  0.67  0.92  0.35  -  0.53 |
| *Continuous variables* | *Median (IQR)* | *Median (IQR)* |  | *Median (IQR)* | *Median (IQR)* |  |
| WPI | 1.5 (0;2.5) | 4 (1;7) | 0.06 | 1 (0;2) | 5.5 (2.5;7) | **0.01** |
| SSS | 2.5 (1.5;4) | 6 (4;7) | **0.001** | 3.5 (3;4) | 6 (5;8) | **0.008** |
| MFI | 46 (40;51) | 70 (62;71) | **<0.001** | 44 (36;57) | 66 (61;81) | **0.005** |
| SF-36 | 70.6 (54.4;79.8) | 52.1 (33.1;63) | **0.01** | 72.1(60;89) | 49.5 (31.1;56.2) | **0.008** |
| Age (yrs) | 54 (44.5;66) | 53 (40;58) | 0.61 | 56 (45;72) | 62.5 (55.5;69.5) | 0.60 |
| Duration | 2.5 (0.5;5.5) | 5 (0;6) | 0.73 | 1.5 (1;3) | 1 (1;5) | 0.88 |
| BMI (kg/m^2^) | 27 (24.7;31.0) | 28.7 (23;38.4) | 0.63 | 28.9 (25.6;30.1) | 27 (24.8;31.2) | 0.56 |
| VDI | 1.5 (0.5;2) | 2 (1;4) | 0.07 | 1.5 (1;3) | 2 (1.5;3) | 0.33 |
| CRP | 1.15 (0.6;3.4) | 5.3 (3.7;24.7) | **0.03** | 4.8 (0.8;8.5) | 3.35 (1.25;5.7) | 0.79 |
| Sleep Disturb. score | 6 (4;7.5) | 6.5 (4;13) | 0.50 | 6 (4;7) | 10.5 (6.5;16.5) | **0.02** |
| Anxiety score | 5 (4;6) | 5 (5;10) | 0.19 | 5.5 (4;6) | 7.5 (3.5;12) | 0.16 |
| Depression score | 3.5 (1.5;5.5) | 6 (3;8) | **0.03** | 3.5 (2;6) | 5.5 (5;9) | 0.12 |
| Cognitive Fail. score | 20.5 (16;26.5) | 43 (21;46) | **0.01** | 29 (25;40) | 48.5 (31;65.5) | 0.14 |

**Legend:** AAV patients n=45, due to the exclusion of nine atypically fatigue patients as defined by the DSQ-2**.**

**Abbreviations:** Y-Yes; F-Female; GPA-Granulomatosis with polyangiitis; MPA-Microscopic polyangiitis; EGPA-Eosinophilic granulomatosis with polyangiitis; VDI-Vasculitis damage index; BMI-Body mass index; CRP-C reactive protein; WPI-Widespread pain index; SSS-Symptom severity score; SF36-Short form 36; MFI-Multidimensional fatigue inventory

**Supplementary Table 3. Pairwise correlations for PR3-ANCA patients**

|  | MFI | Age | CRP | VDI | ENT | Eye | Arthralgia | PSQI | CFQ | HADS  Depression | HADS  Anxiety | SF36 | WPI |
| --- | --- | --- | --- | --- | --- | --- | --- | --- | --- | --- | --- | --- | --- |
| MFI | 1 |  |  |  |  |  |  |  |  |  |  |  |  |
| Age | 0.02 | 1 |  |  |  |  |  |  |  |  |  |  |  |
| CRP | 0.45 | 0.23 | 1 |  |  |  |  |  |  |  |  |  |  |
| VDI | 0.30 | 0.09 | 0.07 | 1 |  |  |  |  |  |  |  |  |  |
| ENT | 0.18 | -0.33 | 0.08 | 0.08 | 1 |  |  |  |  |  |  |  |  |
| Eye | 0.16 | 0.06 | 0.03 | 0.51 | 0.15 | 1 |  |  |  |  |  |  |  |
| Arthralgia | 0.20 | -0.34 | 0.17 | 0.29 | 0.27 | 0.23 | 1 |  |  |  |  |  |  |
| PSQI | 0.28 | -0.30 | -0.16 | -0.09 | 0.20 | -0.22 | -0.02 | 1 |  |  |  |  |  |
| CFQ | 0.54 | -0.23 | 0.39 | -0.10 | 0.20 | 0.05 | 0.15 | 0.49 | 1 |  |  |  |  |
| HADS_Dep | 0.68* | -0.08 | 0.23 | 0.22 | 0.11 | -0.11 | 0.04 | 0.24 | 0.19 | 1 |  |  |  |
| HADS_Anxiety | 0.48 | -0.15 | 0.02 | 0.14 | 0.16 | 0.05 | 0.203 | 0.55 | 0.35 | 0.49 | 1 |  |  |
| SF36 | -0.66* | -0.23 | -0.38 | -0.26 | 0.00 | -0.04 | -0.14 | -0.13 | -0.32 | -0.51 | -0.28 | 1 |  |
| WPI | 0.33 | 0.19 | 0.25 | 0.00 | -0.08 | 0.07 | 0.20 | -0.02 | 0.29 | -0.09 | -0.22 | -0.46 | 1 |

*** p=<0.05**

**** p=<0.01**

*****p=<0.001**

**Abbreviations:** VDI-Vasculitis damage index; ENT-Ear, Nose and Throat; CRP-C reactive protein; PSQI-Pittsburgh sleep quality index; CFQ-Cognitive failures questionnaire; WPI-Widespread pain index; SF36-Short form 36; MFI-Multidimensional fatigue inventory

**Supplementary Table 4. Pairwise correlations for MPO-ANCA patients**

|  | MFI | Age | CRP | VDI | ENT | Eye | Arthralgia | PSQI | CFQ | HADS  Depression | HADS  Anxiety | SF36 | WPI |
| --- | --- | --- | --- | --- | --- | --- | --- | --- | --- | --- | --- | --- | --- |
| MFI | 1 |  |  |  |  |  |  |  |  |  |  |  |  |
| Age | 0.01 | 1 |  |  |  |  |  |  |  |  |  |  |  |
| CRP | 0.06 | -0.14 | 1 |  |  |  |  |  |  |  |  |  |  |
| VDI | 0.01 | 0.20 | 0.16 | 1 |  |  |  |  |  |  |  |  |  |
| ENT | 0.43 | -0.04 | 0.20 | 0.29 | 1 |  |  |  |  |  |  |  |  |
| Eye | -0.08 | 0.21 | -0.02 | 0.26 | -0.03 | 1 |  |  |  |  |  |  |  |
| Arthralgia | 0.18 | 0.06 | 0.05 | 0.04 | 0.28 | 0.01 | 1 |  |  |  |  |  |  |
| PSQI | 0.76** | 0.14 | -0.02 | -0.15 | 0.28 | -0.01 | 0.23 | 1 |  |  |  |  |  |
| CFQ | 0.46 | 0.31 | 0.05 | -0.14 | 0.29 | 0.09 | 0.28 | 0.54 | 1 |  |  |  |  |
| HADS_Dep | 0.73** | -0.00 | 0.16 | -0.25 | 0.23 | -0.01 | -0.08 | 0.63* | 0.49 | 1 |  |  |  |
| HADS_Anxiety | 0.24 | -0.03 | 0.34 | 0.01 | 0.01 | -0.06 | -0.00 | 0.42 | 0.29 | 0.49 | 1 |  |  |
| SF36 | -0.72** | -0.12 | -0.20 | -0.17 | -0.35 | -0.32 | -0.10 | -0.58 | -0.40 | -0.60 | -0.33 | 1 |  |
| WPI | 0.61 | 0.31 | -0.17 | -0.07 | 0.08 | 0.10 | -0.12 | 0.40 | 0.50 | 0.50 | -0.01 | -0.49 | 1 |

*** p=<0.05**

**** p=<0.01**

*****p=<0.001**

**Abbreviations:** VDI-Vasculitis damage index; ENT-Ear, Nose and Throat; CRP-C reactive protein; PSQI-Pittsburgh sleep quality index; CFQ-Cognitive failures questionnaire; WPI-Widespread pain index; SF36-Short form 36; MFI-Multidimensional fatigue inventory

**Supplementary Figure 1. Pain distribution in fatigued PR3-ANCA and MPO-ANCA patients, as compared to fibromyalgia controls**

**
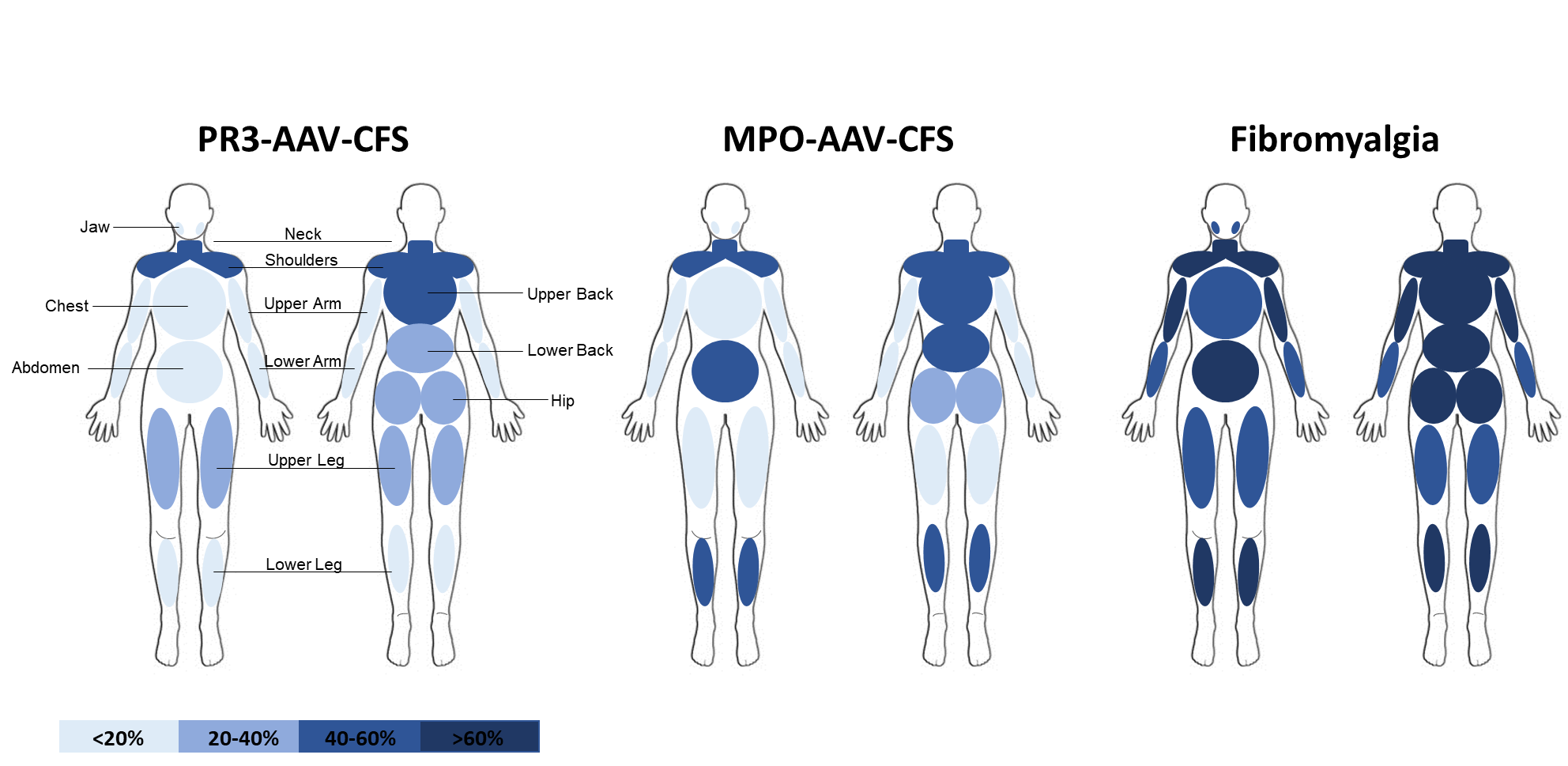
**
